# Gigantic biatrial myxoma in a young female with an atrial septal defect: a case report

**DOI:** 10.1093/ehjcr/ytae197

**Published:** 2024-04-18

**Authors:** Caroline Radner, Linda Grefen, Alexey Dashkevich, Christian Hagl, Christoph S Mueller

**Affiliations:** Department of Cardiac Surgery, LMU Hospital, Marchioninistrasse 15, 81377 Munich, Germany; Department of Cardiac Surgery, LMU Hospital, Marchioninistrasse 15, 81377 Munich, Germany; Department of Cardiac Surgery, LMU Hospital, Marchioninistrasse 15, 81377 Munich, Germany; Department of Cardiac Surgery, LMU Hospital, Marchioninistrasse 15, 81377 Munich, Germany; Department of Cardiac Surgery, LMU Hospital, Marchioninistrasse 15, 81377 Munich, Germany

**Keywords:** Myxoma, Biatrial, Cardiac neoplasm, Cardiac surgery, Case report

## Abstract

**Background:**

Myxomas are uncommon and benign cardiac neoplasms that can present with various cardiac, systemic, embolic, or without symptoms depending on their location and size. Very few cases of large, truly biatrial, or tumours connected via the cardiac atria have been reported throughout the years.

**Case summary:**

We present an unusual case of an apparently healthy 25-year-old French woman, who presented with dyspnoea at Munich’s Octoberfest. Echocardiography and computed tomography identified gigantic masses in left and right atrium, which were connected through an atrial septal defect. They were successfully removed by emergent cardiac surgery.

**Discussion:**

This case describes an uncommon tumour and highlights the importance of a quick diagnosis and prompt surgery. We describe the management and surgery for atrial myxomas as well as demonstrating pre- and intraoperative pictures.

**Learning points**

- Although being a rare disease, cardiac neoplasms should be suspected in an otherwise healthy patient who presents with obstructing ventricular filling and symptoms like dizziness, fatigue, and dyspnoea.
- The need for surgical resection at the appropriate time for extensive intracardiac masses should be recognized.

## Introduction

Myxomas are benign cardiac tumours and, although rare, they account for 50% of intracardiac masses.^1,2^ With an incidence of only 0.0017% among the general population, they mostly affect women. About 75–83% originate from the left atrium, 13–18% from the right and the rest forms in the ventricles. Multiple myxomas occur in <2.5% of cases, with even fewer having a true, bilateral origin.^1–3^

Patients usually present with embolisms, mitral valve obstructions, and sometimes atrial fibrillation. Dyspnoea, palpitations, or dizziness are common symptoms, which depend on the size and location of the myxoma. Early diagnosis and surgical excision are essential in order to prevent embolization and sudden death.^2,4^

Here, we report the case of a young female presenting with massive biatrial myxomas and discuss the subsequent surgical removal as well as the restoration of the septum with a bovine pericardial patch.

## Summary figure

Pre-operative imaging of the biatrial myxoma (*A*: transthoracic echocardiography, apical four-chamber view, *B*: transthoracic echocardiography, parasternal long axis view; red arrows indicate myxoma), *C*: excised myxoma with connecting stalk.

**Figure:**
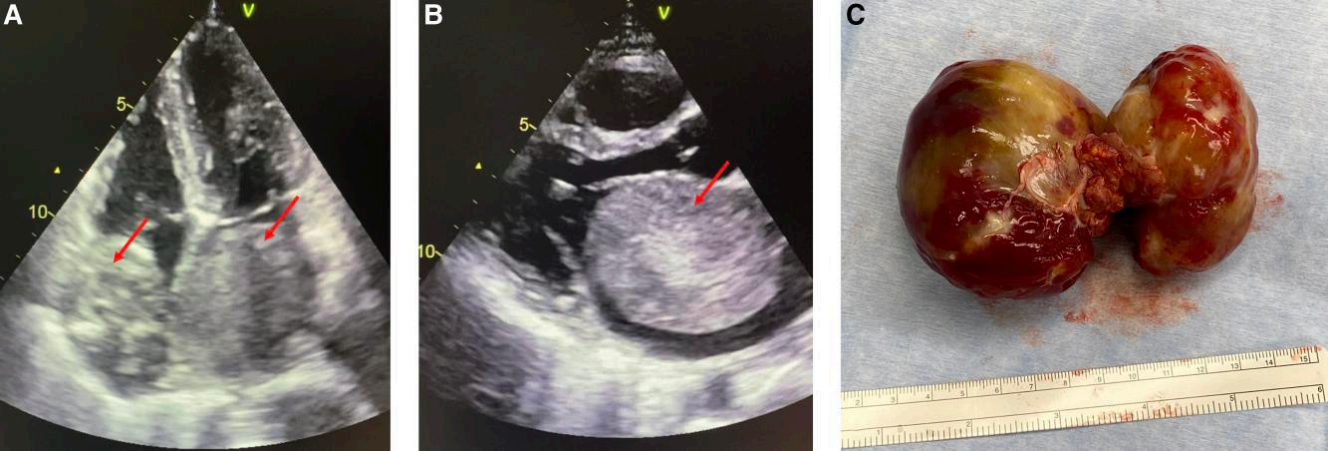


## Case presentation

A 25-year-old French, Caucasian woman presented to the emergency room with an acute episode of dyspnoea at Munich’s Octoberfest. Upon questioning, the patient reported occasional fatigue in the weeks preceding the event but no exertional dizziness, history of syncope, or constitutional symptoms like fever.

Physical examination showed lung auscultation clear anteriorly and posteriorly, minor sinus tachycardia (112 beats/min) with no extra heart sounds or murmurs as well as normothermia (36.5°C), and regular blood pressure (108/52 mmHg). The patient reported being generally fit and well, with no regular medication use or smoking habits, and her family history did not reveal any notable illnesses.

**Figure 1:**
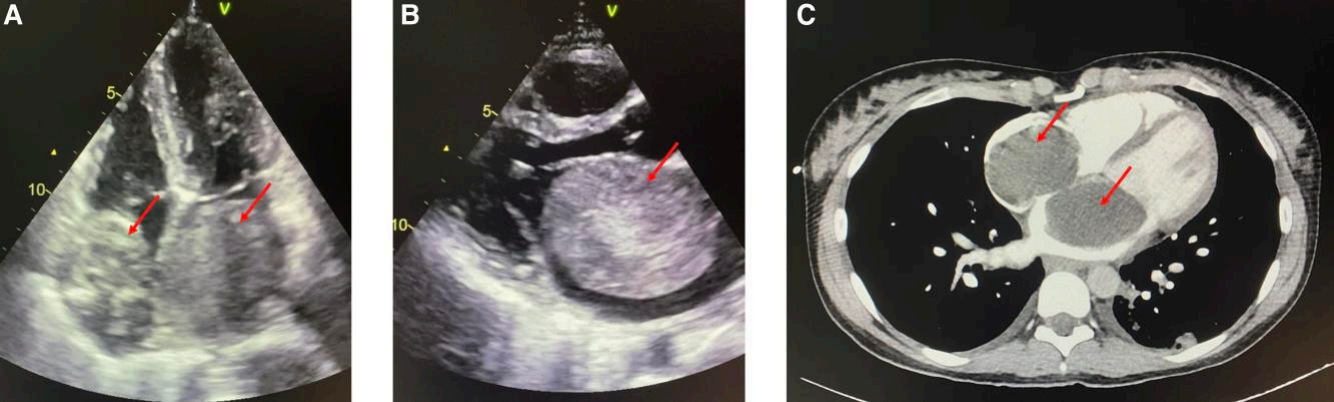
(*A*–*C*) Pre-operative imaging of the biatrial myxoma. (*A*, *B*) Transthoracic echocardiography (*A*: apical four-chamber view, *B*: parasternal long axis view), (*C*) CT scan, the arrows indicate the myxoma.

Further laboratory workup revealed an elevated C-reactive protein (4 mg/dL) and raised liver function tests (GOT 70 U/L, GPT 72 U/L) as well as slightly increased NT-proBNP (1200 pg/mL) and leucocytes (15.5G/L). Electrocardiogram was normal, but an echocardiography and a CT scan (see *Figure 1A–C*) revealed a massive tumour extending into both atria, measuring 8 × 5 cm on the left and 5 × 4 cm on the right side interfering with mitral and tricuspid valve function resulting in a minor, functional mitral stenosis as well as minimal mitral and tricuspid valve insufficiency and therefore obstructing ventricular filling. Due to the significant findings, the patient underwent surgery the following morning.

For surgical excision, the patient was cannulated via the right femoral vein as well as the superior vena cava and ascending aorta. Cardiopulmonary bypass was commenced, and the heart arrested with cold Bretschneider cardioplegia.

**Figure 2:**
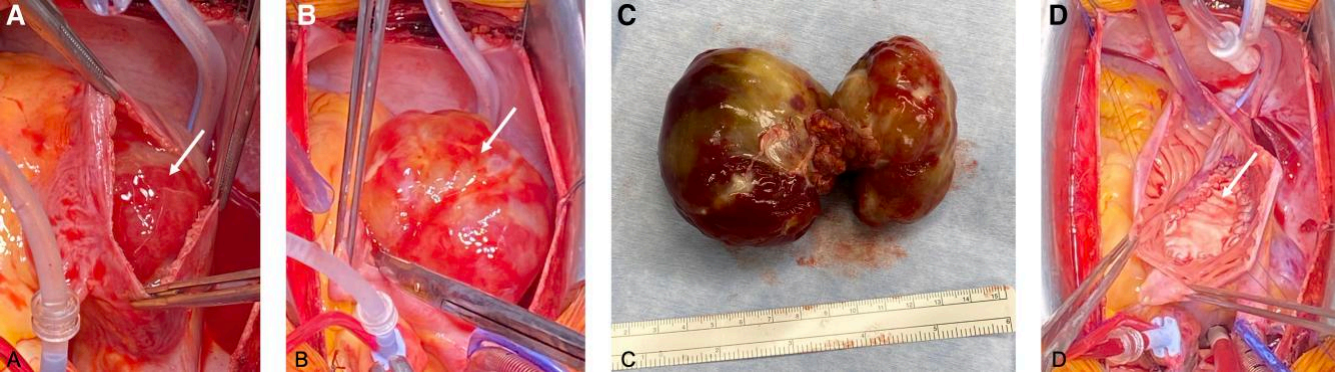
(*A*–*D*) Intraoperative images. (*A*, *B*) Revelation of the myxoma after incising the right atrium, (*C*) myxoma after excision, including connecting stalk, (*D*) ASD repair with bovine pericardial patch.

The incision of the right atrium showed a mass filling the whole atrium (see *Figure 2A* and *B*) and adhering to the septum as well as the fossa ovalis. The left atrium was inspected transseptally and showed the rest of the sizable tumours. The septum was circumferentially excised in order to easily mobilize and remove the myxoma (see *Figure 2C*). The resulting atrial septal defect (ASD) was closed with a bovine pericardial patch using a 4-0 prolene running suture (see *Figure 2D*).

The post-operative course proceeded without any complications and the patient displayed normal sinus rhythm without the need of a permanent pacemaker. She was extubated 14 h after surgery, stayed in the ICU for 2 days, and received ampicillin and azithromycin to treat a hypostatic pneumonia, which was thought to be caused by pulmonary congestion due to the myxoma. The final pathology report revealed that the mass was, indeed, myxoid tissue, with metaplastic ossification but no signs of malignancy. The patient was discharged from the hospital after 10 days without any residual symptoms of dyspnoea. Upon routine follow-up via telephone 3 months later, she also reported the disappearance of the occasional fatigue that was mentioned in the weeks before the diagnosis and surgery.

## Discussion

As previously mentioned, cardiac myxomas can present with symptoms mimicking various cardiovascular conditions, including palpitations, chest pain, dyspnoea, and syncope as well as constitutional and systemic symptoms such as fever, fatigue, and even weight loss. This case underscores the diagnostic challenges associated with myxomas. Prompt treatment at the time of diagnosis is essential to prevent life-threatening events, like embolisms and neurologic sequelae. Echocardiography may not always provide a definitive diagnosis in cases of smaller myxomas, and a high index of suspicion is crucial for timely intervention. To reach a diagnosis, collaboration with an interdisciplinary team involving cardiologists, radiologists, and cardiothoracic surgeons is essential to arrive at a conclusive assessment. Detailed imaging studies, including transoesophageal echocardiography and cardiac CT or MRI, play a pivotal role, as demonstrated in this particular case. In accordance with the 2022 ESC Guidelines on cardio-oncology, myxomas should primarily be treated with surgical resection, especially if they are prone to embolize or cause obstruction.^5^ The primary treatment for cardiac myxomas is surgical excision. The tumour is to be carefully removed, and efforts are to be made to preserve surrounding cardiac structures. Intraoperative transoesophageal echocardiography is often employed to ensure complete removal of the tumour and to assess the overall cardiac function. Examination of the excised tumour tissue is crucial for confirming the diagnosis and determining the nature of the myxoma. Cardiac myxomas exhibit a characteristic histopathology, featuring a myxoid matrix with stellate or spindled cells within a mucopolysaccharide-rich stroma. The tumour is typically benign, showing low mitotic activity, presence of blood vessels, and occasional calcification, while immunohistochemistry is positive for endothelial markers such as CD31 and CD34.^6^

After surgical excision, prognosis is excellent, but regular echocardiography follow-ups are necessary to detect recurrence. Biatrial myxomas are rare and usually originate from the limbus of the fossa ovalis. Usually, they share a common attachment site on opposite sides of the interatrial septum, as suspected in our case. The first successful removal of a biatrial myxoma was described in 1967 by Yipintsoi *et al*. where they reported the case of a 29-year-old female with a large right atrial myxoma extending through the septum and a small myxoma on the free wall of the left atrium.^3^ Going forward, there were more cases reported, but the tumours mostly expressed two vastly different sizes with one atrium inhabiting a comparably smaller myxoma, or two separate masses not connected via the atrial septum. This differs from the case we describe above, where one sizable mass grew from one atrium via an ASD into the opposite side of the heart.^4,7^

This case report underscores the intricate nature of diagnosing and managing cardiac myxomas. It emphasizes the need for a multidisciplinary approach, advanced imaging modalities, and vigilant post-operative care. Sharing such clinical lessons enhances our understanding of rare cardiac pathologies and contributes to improved patient outcomes.

## Data Availability

The data underlying this article are available in the article and upon contacting the corresponding author.

## Lead author biography

Dr Caroline Radner is a cardiac surgery resident at Ludwig-Maximilian University in Munich, Germany. She graduated from Paracelsus Medical School in Salzburg and received the MD degree in 2020. She is interested in cardiac surgery as well as interventional cardiology.
